# Effect of pumpkin on the quality characteristics and storage quality of aerobically packaged chicken sausages

**DOI:** 10.1186/2193-1801-3-39

**Published:** 2014-01-21

**Authors:** Fayaz Ahmed Zargar, Sunil Kumar, Zuhaib Fayaz Bhat, Pavan Kumar

**Affiliations:** Veterinary Officer, Mirah Exports Pvt Ltd, Dera Bassi, Sas Nagar, Punjab 144410 India; Division of Livestock Products Technology, Faculty of Veterinary Sciences and Animal Husbandry, Sher-e-Kashmir University of Agricultural Sciences and Technology of Jammu, R. S. Pura, Jammu, Jammu and Kashmir 181102 India; Division of Livestock Products Technology, GADVASU, Ludhiana, Punjab 141004 India

**Keywords:** Chicken sausages, Pumpkin, Crude fibre, Refrigerated storage

## Abstract

The present study was undertaken to evaluate the effect of different levels of pumpkin on the quality characteristics of chicken sausages. The pumpkin was incorporated at three different levels viz. 6, 12 and 18 percent replacing lean meat in the formulation. The products were analyzed for various physicochemical and sensory attributes. pH, emulsion stability, cooking yield, crude protein, ether extract and ash content of the products showed significantly (p < 0.05) decreasing trend with increasing levels of incorporation of pumpkin however, there was a significant (p < 0.05) increase in the moisture and crude fibre content. Based on various parameters, 12 percent level of incorporation was optimized as best. Chicken sausages with optimum level of pumpkin along with control were aerobically packaged in LDPE pouches and assessed for storage quality under refrigerated (4 ± 1°C) conditions. The mean values of pH and all the sensory parameters showed significantly (p < 0.05) decreasing trend for both control as well as treatment samples whereas TBARS (mg malonaldehyde/kg) value, total plate count (log cfu/g) and yeast and mould count (log cfu/g) showed significantly (p < 0.05) increasing trend with storage. Coliforms (log cfu/g) were not detected throughout the period of storage. Thus, fibre enriched chicken sausages could be successfully stored for a period of 14 days at refrigeration temperature (4 ± 1°C) without any significant loss in quality.

## Introduction

Studies have proven a relationship between a diet containing an excess of energy-dense foods and the emergence of a range of chronic diseases including colon cancer, obesity, cardiovascular diseases, and several other disorders (Bhat et al., 2011; Biswas et al., 2011; Tungland and Meyer, 2002; Beecher, 1999). Sadri and Mahjub (2006) reported a positive association between meat consumption and colorectal cancer. Therefore, an increase in the level of dietary fiber in the daily diet has been recommended (Bhat and Bhat, 2011a, 2011b; Johnson and Southgate, 1994; Eastwood, 1992,). Dietary fibres are the remnants of the edible part of plants and analogous carbohydrates that are resistant to digestion and absorption in the human small intestine (Prosky, 1999). Dietary fibres are the key ingredient lacking in the meat and meat products and regular consumption of latter is being associated with various health disorders. Various reports have revealed that intake of fibre reduces the risk of such diseases. Several studies have proven that dietary fibers have the potential to reduce blood low density lipoprotein cholesterol (Brown et al., 1999), risk of diabetes mellitus type 2 (Willet et al., 2002), coronary heart disease (Bazzano et al., 2003), blood pressure (Streppel et al., 2005), obesity (Liu, et al., 2003) and colorectal cancer (Schatzkin et al., 2007; Tharanathan and Mahadevamma, 2003).

Studies have shown that the consumption of fruits and vegetables imparts the dietary fiber and health benefits (Schieber et al., 2001). There has been a dramatic shift in consumer eating habits in recent years. As consumers have become increasingly more health conscious, the trend is toward foods including meat products with decreased levels of fat, salt, cholesterol and caloric content as well as enriched with dietary fibre (Yang et al., 2007). With increasing awareness consumers try to reduce dietary calorie intake by increasing dietary fibre in the diet (Bhat and Bhat, 2011a, 2011b). Manufactures also have responded and introduced several modifications by incorporating various vegetable and fruit fibers in the formulations in an attempt to offset the detrimental effects of energy dense foods.

Besides having many health benefits, fiber improves many technological properties of the products. Fiber is suitable for addition to meat products and has previously been used in cooked meat products to increase the cooking yield due to its water-binding and fat-binding properties and to improve texture (Cofrades et al., 2000). Various types of fiber have been studied alone or combined with other ingredients for formulations of meat products (Bhat and Bhat, 2011a, 2011b; Bhat and Pathak, 2009; Yilmaz, 2004; Modi et al., 2003; García et al., 2002). Many vegetable fiber sources have been utilized in the development of fiber fortified meat products, however, very meagre information is available on the utilization of pumpkin as a source of dietary fibre in the development of meat products. The utilization of pumpkin in the development of meat products is still an unexplored area.

Pumpkin (*Cucurbita maxima*), a member of the cucurbitacae family, is grown under a wide range of agro climatic conditions in India both for immature and mature fruits. It is considered as the marvel of the vegetable world due to its unusual and extravagant characteristics. When ripe, the fruit is sweet with yellow or orange flesh and valuable source of carotenoids and ascorbic acid (Sirohi et al., 1991). Besides, being nutritionally rich, pumpkin also possess many medicinal properties. The pulp of the fruit is considered as sedative, emollient and refrigerant (Bhat et al., 2010a).

India has made a spectacular progress in the poultry sector from last few decades and has emerged as a fastest growing sector in livestock economy. Chicken meat is considered relatively leaner and there is no social taboo attached to its consumption, making it the most preferred meat in India. Furthermore, the rising costs of chevon and mutton in India coupled with requirement to develop convenient meat items have created a need for alternatives. Thus, prospects of further developing certain chicken meat products, like sausages, by extending with certain low cost fibre sources, like pumpkin, could find increasing popularity in food service industry particularly at fast food outlets.

Keeping in view all the above facts the present study was envisaged to attempt the still inconclusive studies on utilization of pumpkin in the development of fibre fortified meat products. A study was designed to evaluate the effect of different levels of pumpkin pulp on physicochemical properties and sensory profile of chicken sausages and to assess the storage quality of the developed products.

## Material and methods

### Chicken meat

The broiler birds of 6 weeks of age were procured from state animal husbandry department and slaughtered by the *Halal* method in the Division of Livestock Products Technology, SKUAST-J. The body fat was trimmed and deboning of dressed chicken was done manually by removing all the tendons and separable connective tissue. The lean meat was packed in polythene bags and stored under frozen conditions at -18 ± 2°C until use.

### Fat

Refined cottonseed oil of brand name ‘*Sheerji*’ was purchased from local market and used in emulsion preparation.

### Condiment mixture

Condiments used were fresh onion, garlic and ginger. The external covering of all were peeled off and cut into pieces. The cut pieces were weighed in a ratio of 3:2:1 and ground in a mixer to the consistency of fine paste.

### Spice mixture

The spice mixture was standardized and developed in the laboratory and contained anise (*Pimpinalla anisum*, *soanf*-13%), bay leaves (*Laurus nobilis*, *tej patta*-2%), black pepper (*Piper nigrum*, *kali mirch*-5%), green cardamom (*Elettaria cardamomum*, choti elaichi-5%), cinnamon (*Cinnamomum zeylanicum*, *dalchini*-6%), cloves (*Syzygium aromaticum*, *laung*-2%), dry fenugreek powder (*Foenum-graecum*, *meathi*-6%), coriander (*Coriandrum sativum*, *dhania*-20%), cumin seed (*Cuminum cyminum*, *jeera*-12%), mace (*Myristica fragrans*, *javitri*-2%), nutmeg (*Myristica fragrans*, *jaiphal*-2%), red chilli (*Capsicum frutescens*, *lal mirch*-12%), black cardamom (*Amomum subulatum*, *badi elaichi*-5%), mint leaves (*Lamiaceae*, *pudina*-3%) and dry ginger powder (*Zingiber officinale*, *saunth*-5%).

### Pumpkin

Vegetable used in the study as a source of fiber viz. mature pumpkin was sourced from local market of Jammu and its pulp was incorporated at different levels i.e. 0, 6, 12, and 18 percent in the formulation by replacing lean meat.

### Method of preparation of chicken sausages

Several preliminary trials were conducted to optimize the basic formulation and processing conditions for the preparation of chicken sausages.

### Preparation of emulsion

The standardized formulation for the preparation of emulsion for chicken sausages is presented in Table 1. Lean meat from dressed chicken was cut into smaller chunks and minced in a Sirman mincer (MOD-TC 32 R10 U.P. INOX, Marsango, Italy) with 6 mm plate. Meat emulsion was prepared in Sirman Bowl Chopper [MOD C 15 2.8G 4.0 HP, Marsango, Italy]. Minced meat was blended with salt, sodium tripolyphosphate and sodium nitrite for 1.5 minute. Water in the form of crushed ice was added and blending continued for 1 minute. This was followed by the addition of refined vegetable oil and blended for another 1 to 2 minutes. This was followed by addition of spice mixture, condiments and other ingredients and again mixed for 1 to 2 minutes to get the desired emulsion.

**Table 1:** **The standardized formulation for the preparation of emulsion for chicken sausages**

Lean meat	64.45%
Added water	10%
Vegetable oil	10%
Condiment mixture	5%
Refined wheat flour	3%
Whole egg liquid	3%
Spice mixture	2%
Table salt	1.75%
Monosodium glutamate	0.5%,
Sodium tripolyphosphate	0.3%
Sodium nitrite	120 ppm

### Stuffing of casings

The emulsion was filled into the Sirman sausage filler (Model-1S-V15-IRDA-VERT, S.No. 07 L01410. Marsango, Italy) and the artificial cellulose casing was applied on the nozzle of sausage filler. Pressure was applied in such a way so that the emulsion was filled into the casings.

### Cooking of raw chicken sausages

Preliminary trials were conducted to optimize the processing conditions for the preparation of chicken sausages. The raw sausages were cooked at different time-temperature combinations and based on mean scores of various sensory parameters, cooking at a temperature of 140 ± 5°C for 25 minutes was found to be optimum. Raw sausages were hung vertically on skewers which were placed at the ridges along the two ends of hot air oven (Yorco sales Pvt. Ltd. India, Model-YS1-431, S. No. 02B2843). Raw sausages were cooked in a preheated hot air oven at 140 ± 5°C for a total time of about 25 minutes. The internal temperature of sausages was monitored by a thermometer and cooked to an internal temperature of 80 ± 2°C.

### Analytical procedures

#### pH and cooking yield

The pH of cooked chicken sausages was determined by the method of Keller et al. (1974) using a digital pH meter (Systronics Digital pH Meter 803, serial No. 603).

The weight of each sausage was recorded before and after cooking. The cooking yield was calculated and expressed as percentage by a formula:

### Proximate analysis

Moisture, crude protein, crude fat and ash contents in both treatment samples and control were determined by using standard procedures prescribed by AOAC (2000).

### Emulsion stability and thiobarbituric acid reacting substances (TBARS) value

Emulsion stability of meat emulsion was determined as per procedure described by Townsend et al. (1968) with some modifications.

Thiobarbituric acid reacting substances (TBARS) value of cooked products during storage was determined using the method described by Witte et al. (1970).

### Microbiological profile

Total plate count, psychrophillic count, coliform count and yeast and mold count were determined by methods described by APHA (1984). Readymade media (Hi-Media) were used for the analysis.

### Sensory evaluation

The sensory evaluation of the products was carried for various attributes namely colour and appearance, flavour, juiciness, texture and overall acceptability by a panel of seven experienced panellists on a 8-point hedonic scale, wherein 8 denoted “extremely desirable” and 1 denoted “extremely undesirable” (Seman et al., 1987). Panellists were seated in a room free of noise and odours and suitably illuminated. Coded samples for sensory evaluation were prepared and served warm to panellists. Water was provided for oral rinsing between the samples.

### Statistical analysis

Means and standard errors were calculated for different parameters. Data obtained in the study were analysed statistically on ‘SPSS-16.0’ software package as per standard methods (Snedecor and Cochran, 1994). Duplicate samples were drawn for each parameter and the experiment was replicated thrice (n = 6). Sensory evaluation was performed by a panel of seven member judges three times, so total observations being 21 (n = 21). Data were subjected to one way analysis of variance and level of significance among the treatments and two way analysis of variance and level of significance in case of storage study.

The permission for use of poultry birds for the research purpose was taken from the Institutional Animal Ethics Committee (IAEC), SKUAST-Jammu.

## Results and discussion

### Physicochemical characteristics

The mean values of various physicochemical parameters namely pH, cooking yield, emulsion stability and proximate composition of chicken sausages incorporated with 0, 6, 12 and 18 percent levels of pumpkin are presented in Table 2.

**Table 2:** **Effect of pumpkin on pH, cooking yield, emulsion stability and proximate composition of chicken sausages (Mean ± SE)** ^*^

Parameters	Levels of pumpkin (%)			
	0	6	12	18
pH	6.38^**c**^ ± 0.02	6.30^**b**^ ± 0.05	6.25^**b**^ ± 0.01	6.12^**a**^ ± 0.02
Cooking Yield (%)	92.63^**d**^ ± 0.08	92.13^**c**^ ± 0.08	91.20^**b**^ ± 0.13	89.94^**a**^ ± 0.08
Emulsion Stability (%)	91.96^**c**^ ± 0.19	91.86^**c**^ ± 0.24	89.89^**b**^ ± 0.12	89.00^a^ ± 0.25
Moisture (%)	63.11^**a**^ ± 0.26	66.02^**b**^ ± 0.25	66.82^**c**^ ± 0.28	67.63^**d**^ ± 0.07
Crude Protein%)	16.85^**b**^ ± 0.12	16.55^**b**^ ± 0.13	16.14^**a**^ ± 0.02	15.81^**a**^ ± 0.16
Ether Extract (%)	13.78^**bc**^ ± 0.15	13.83^**c**^ ± 0.02	13.56^**b**^ ± 0.02	13.23^**a**^ ± 0.02
Crude Fibre (%)	0.72^**a**^ ± 0.02	0.93^**b**^ ± 0.03	1.11^**c**^ ± 0.02	1.81^**d**^ ± 0.01
Ash (%)	2.81^**d**^ ± 0.02	2.71^**c**^ ± 0.02	2.54^**b**^ ± 0.02	2.36^**a**^ ± 0.02

^*^Mean ± SE with different superscripts in a row differs significantly (P < 0.05), n = 6.

### pH

A significant (P < 0.05) decrease in pH was observed in the products with increase in the level of incorporation of pumpkin which might be attributed to the ascorbic acid content of pumpkin and acid terminal residues in the starch molecules produced by depolymerisation of starch granules during cooking (Perez, 1997). Similar decrease in the pH was reported by Verma et al. (2013) in sheep meat nuggets on incorporation of guava powder and Banerjee et al. (2012) in goat meat nuggets on incorporation of broccoli powder extract. Verma et al. (2012a) and (2012b) also observed a decrease in pH of chicken nuggets incorporated with bottle gourd and chickpea hull flour, respectively.

### Cooking yield

A significantly (P < 0.05) decreasing trend was observed in the cooking yield with increasing levels of pumpkin. This could be due to formation of comparatively less stable emulsion for the formulations containing pumpkin. Additionally lower emulsion stability might have resulted in the loss of moisture content while cooking. Similar decrease in cooking yield was also reported by Verma et al. (2013) in sheep meat nuggets on incorporation of guava powder, Banerjee et al. (2012) in goat meat nuggets on incorporation of broccoli powder extract, Verma et al. (2012a) in low-fat chicken nuggets on sodium chloride replacement and incorporation of bottle gourd and by Verma et al. (2012b) in low-fat chicken nuggets on sodium chloride replacement and added chickpea hull flour. Similar decrease in the cooking yield was also reported by Verma et al. (2010) in low-fat chicken nuggets on sodium chloride replacement and apple pulp inclusion and Devatkal et al. (2004) in buffalo loaves on incorporation of carrot and potato.

### Emulsion stability

The emulsion stability of the raw products followed a decreasing trend with increasing levels of pumpkin in the formulation. This decrease was significantly (P < 0.05) lower at 12 and 18 percent level of incorporation in comparison to control. The probable reasons for the decreased emulsion stability due to pumpkin inclusion could be lowering of the pH value of emulsion, poor fat binding capacity of pumpkin and interference in the formation of uniform and stable emulsion. Verma et al. (2013) reported similar findings in sheep meat nuggets on incorporation of guava powder. A similar decrease in emulsion stability was also reported by Banerjee et al. (2012), Verma et al. (2012a), (2012b) and (2010) in various meat products.

### Proximate composition

#### Moisture

A significant (p < 0.05) increase in moisture percent was recorded with increase in each level of pumpkin. The moisture content was highest at 18 percent level and it differed significantly (p < 0.05) with all other levels of incorporation. This increase in moisture content might be due to higher moisture present in pumpkin. Suradkar et al. (2013) and Das et al. (2013) also reported an increase in the moisture content of chicken nuggets containing bread crumbs and chicken nuggets containing fermented bamboo shoot, respectively. This was in agreement with the findings of Verma et al. (2010) who also observed a similar finding in designer chicken nuggets incorporated with apple pulp.

#### Protein

The crude protein content of chicken sausages decreased significantly (P < 0.05) with lean meat replacement, however, reduction was not significant (P > 0.05) between the variants prepared by incorporation of 12 and 18 percent levels of pumpkin. At 18 percent level it was significantly (P < 0.05) lower as compared to control. The probable reasons for the decreased protein content may be attributed to the comparatively lower protein contents of pumpkin pulp. Suradkar et al. (2013) also reported a decrease in the protein content of chicken nuggets containing bread crumbs. Verma et al. (2013) also observed a decrease in the protein content of sheep meat nuggets on incorporation of guava powder. Taludkar and Sharma (2009) observed a decrease in protein content of chicken meat patties incorporated with wheat and oat bran. Similar results were reported by Candogan (2002) for beef patties with added tomato paste and by Anderson and Berry (2001) for high-fat ground beef with added inner pea fibre.

#### Crude fat

A gradual decrease in ether extract was recorded and was significantly (P < 0.05) low at 18 percent level as compared to control and other levels of pumpkin. Verma et al. (2013) also observed a decrease in the fat content of sheep meat nuggets on incorporation of guava powder. Suradkar et al. (2013), Verma et al. (2012a) and (2012b) also reported similar results in different meat products. Taludkar and Sharma (2009) observed a decrease in fat content of chicken meat patties incorporated with wheat and oat bran. Similar results were reported by Valeria et al. (2008) for dry fermented sausages prepared by the incorporation of carrot dietary fibres and Aleson-carbonell et al. (2004) for non-fermented dry-cured sausages formulated with lemon albedo.

#### Ash

A significantly (P < 0.05) lower value for ash was observed at all incorporation levels as compared to control. Similar results were reported by Verma et al. (2010) for low fat chicken nuggets prepared by the incorporation of apple pulp.

#### Crude fiber

The crude fibre content increased significantly (P < 0.05) with increasing levels of pumpkin. This increase in crude fibre might be due to high fibre level present in pumpkin. Similar increase in the fiber content was also observed by Verma et al. (2013), Das et al. (2013), Verma et al. (2012a), (2012b) and Taludkar and Sharma (2009) in various meat products.

#### Sensory attributes

The mean values of various sensory parameters namely appearance and colour, flavour, juiciness, texture and overall acceptability of chicken sausages incorporated with 0, 6, 12 and 18 percent levels of pumpkin are presented in Table 3.

**Table 3:** **Effect of pumpkin on sensory attributes of chicken sausages (Mean ± SE)** ^*^

Sensory attributes	Levels of pumpkin (%)			
	0	6	12	18
Appearance & colour	6.90 ± 0.11	7.04 ± 0.03	6.95 ± 0.12	6.80 ± 0.13
Flavour	7.04 ± 0.03	7.19 ± 0.10	7.28 ± 0.08	7.11 ± 0.13
Juiciness	6.90^**a**^ ± 0.11	6.97^**a**^ ± 0.13	7.02^**ab**^ ± 0.02	7.28^**b**^ ± 0.08
Texture	7.23^**b**^ ± 0.08	7.19^**b**^ ± 0.19	7.35^**b**^ ± 0.09	6.33^**a**^ ± 0.10
Overall acceptability	6.47^**a**^ ± 0.11	6.88^**b**^ ± 0.12	7.40^**c**^ ± 0.09	6.52^**ab**^ ± 0.16

^*^Mean ± SE with different superscripts in a row differs significantly (p < 0.05). Mean values are scores on 8 point descriptive scale where 1- extremely poor and 8- extremely desirable.

No significant (P > 0.05) effect of pumpkin was observed on the appearance and colour and flavor scores of the chicken sausages. These observations were in agreement with the findings of Verma et al. (2013) who also observed a non-significant (P > 0.05) effect of guava powder on the appearance and colour and flavor scores of sheep meat nuggets. Suradkar et al. (2013) also reported a non-significant (P > 0.05) effect of bread crumbs on the appearance and colour and flavor scores of chicken nuggets. Banerjee et al. (2012) observed similar findings in goat meat nuggets on incorporation of broccoli powder extract.

The mean juiciness scores showed a significantly (P < 0.05) increasing trend with increasing levels of incorporation of pumpkin. The mean juiciness scores were significantly (P < 0.05) higher for products containing 18 percent level of pumpkin than 6 percent level and control, although, it was comparable (P > 0.05) with 12 percent level of incorporation. Higher juiciness scores in the products with increase in the level of pumpkin could be possibly due to high moisture content of pumpkin pulp. An increase in moisture levels has been reported to increase juiciness in beef patties (Serdaroglu, 2006), goat patties (Gujral et al., 2002) and frankfurters (Hung and Carpenter, 1997). The mean texture scores were significantly (P < 0.05) lower for products containing 18 percent level of incorporation as compared to rest of the treatments and control. The score for overall acceptability increased significantly (P < 0.05) up to 12 percent level and thereafter declined at 18 percent level of incorporation of pumpkin. Similar results were reported by Suradkar et al. (2013) in chicken nuggets and Serdaroglu (2006) in beef patties.

Thus, based on various sensory and physicochemical parameters, 12 percent level of incorporation of pumpkin was optimized as best and was taken up for storage (4 ± 1°C) studies within LDPE pouches along with control.

#### Storage studies

The sausages prepared with the incorporation of 12 percent pumpkin and control were packed aerobically in low density polyethylene (LDPE) pouches and were kept at refrigeration temperature (4 ± 1°C). These pouches were opened under hygienic conditions at a regular interval of 0, 7, 14 and 21 days (till spoilage) for analyzing the different physicochemical, microbiological and sensory properties.

#### Physicochemical characteristics

The mean values of various physicochemical parameters namely pH, thiobarbituric acid reacting substances (TBARS) value and free fatty acid value of aerobically packaged cooked chicken sausages incorporated with 0 and 12 percent levels of pumpkin during refrigerated storage (4 ± 1°C) are presented in Table 4.

**Table 4:** **Effect of refrigerated storage on pH, thiobarbituric acid reacting substances (TBARS) value and free fatty acid (FFA) value of aerobically packaged cooked chicken sausages (Mean ± SE)** ^*^

Treatments	Storage period (Days)			
	0	7	14	21
**pH**				
**CO (0%)**	6.31 ± 0.06^c^	6.26 ± 0.03^bc^	6.19 ± 0.01^ab^	6.10 ± 0.02^a^
**PK (12%)**	6.27 ± 0.02^c^	6.21 ± 0.01^c^	6.13 ± 0.02^b^	6.01 ± 0.02^a^
**TBARS (mg malonaldehyde/kg.)**				
**CO (0%)**	0.37 ± 0.01^aB^	0.47 ± 0.01^bB^	0.58 ± 0.0^cB^	0.73 ± 0.01^dB^
**PK (12%)**	0.24 ± 0.01^aA^	0.32 ± 0.01^bA^	0.53 ± 0.01^cA^	0.66 ± 0.01^dA^
**Free fatty acid (% oleic acid)**				
**CO (0%)**	0.0022 ± 0.00^a^	0.015 ± 0.001^b^	0.027 ± 0.00^Bc^	0.047 ± 0.001^Bd^
**PK (12%)**	0.0021 ± 0.00^aa^	0.013 ± 0.001^b^	0.022 ± 0.001^Ac^	0.042 ± 0.001^Ad^

^*^Mean ± SE with different superscripts in a row (lower case alphabet) and column (upper case alphabet) differ significantly (p < 0.05), n = 6 for each treatment, CO = Control, PK = Pumpkin.

#### pH

pH of chicken sausages followed a significant (P < 0.05) decreasing trend after 7^th^ day of storage, however, the mean pH values of the control samples were comparable (P > 0.05) with mean pH values of treatment samples throughout the period of storage. The decrease in pH might be attributed to the lactic acid produced from readily available carbohydrate molecules by the microbes (Jay, 1996). The reduction in pH after 7^th^ day of refrigerated storage might be due to growth of psychrophilic gram-positive bacteria especially lactic acid bacteria as reported by Shelef (1975). Singh et al. (2011) and García et al. (2002) also observed a similar decrease in pH of chicken snacks and dry fermented sausages, respectively.

#### Thiobarbituric acid reacting substances (TBARS) value

A significant (P < 0.05) effect of storage period was observed on TBARS values of both control as well as treatment samples. TBARS values followed a significantly (p < 0.05) increasing trend from day 0 to 21 in case of both control as well as treated sausages. The increase in TBARS values on storage might be attributed to oxygen permeability of packaging material (Brewer et al., 1992) that led to lipid oxidation. Similar findings were reported during refrigerated storage by Bhat et al. (2013a) in chicken *seekh kababs*, Bhat et al. (2013b) in chicken meat balls, Kumar and Tanwar (2011) in chicken nuggets, Bhat and Pathak (2011) in mutton *Harrisa*, Bhat et al. (2011) in chicken *seekh kababs*, Sudheer et al. (2010) in restructured chicken block, Chidanandaiah et al. (2009) in meat patties and Modi et al. (2003) in buffalo meat burger. TBARS values of products containing pumpkin increased significantly (P < 0.05) through the period of storage. However, the values of TBARS for products containing pumpkin were significantly (P < 0.05) lower than control samples on all days of storage. A comparatively slow increase in TBARS value of sausages extended with 12 percent pumpkin might be due to lower fat percent and increased fiber and carotenoid content which acted as antioxidant. Similar findings were reported by Bhat et al. (2013a) and (2011) in chicken *seekh kababs* extended with different non-meat proteins. Das et al. (2013) and Banerjee et al. (2012) also reported similar results in chicken nuggets containing fermented bamboo shoot and goat meat nuggets containing broccoli powder extract, respectively. Bertelsen et al. (1991) also reported about the natural antioxidant nature of pea fiber.

#### Free fatty acid (FFA)

FFA value followed a significant (P < 0.05) linear increasing trend from day 0 to 21 in pumpkin extended products as well as control. A difference among the control and treatment also existed on day 14 and 21 of storage. Sausages extended with pumpkin as well as control had comparable FFA values with each other on day 0 and 7 of storage. Similar trend was observed by Bhat et al. (2013a) and (2011) in chicken seekh kebabs extended with different non-meat proteins and Nayak and Tanwar (2004) and Nagamallika et al. (2006) in chicken patties during refrigerated storage.

#### Microbiological characteristics

The mean values of various microbiological parameters namely total plate count, psychrophillic count and yeast and mould counts of aerobically packaged cooked chicken sausages incorporated with 0 and 12 percent levels of pumpkin during refrigerated storage (4 ± 1°C) are presented in Table 5.

**Table 5:** **Effect of refrigerated storage on microbiological characteristics of aerobically packaged cooked chicken sausages (Mean ± SE)** ^*^

Treatments	Storage period (Days)			
	0	7	14	21
**Total plate count (log cfu/g)**				
**CO (0%)**	2.45 ±0.01^a^	2.94 ± 0.01^bA^	3.92 ± 0.01^cA^	4.50 ± 0.01^dA^
**PK (12%)**	2.48 ± 0.02^a^	3.32 ± 0.01^bB^	4.10 ± 0.03^cB^	4.93 ± 0.02^dB^
**Psychrophilic count (log cfu/g)**				
**CO (0%)**	ND	1.68 ± 0.01^aA^	2.14 ± 0.01^bA^	2.90 ± 0.02^cA^
**PK (12%)**	ND	1.82 ± 0.02^aB^	2.53 ± 0.02^bB^	3.15 ± 0.02^cB^
**Yeast and mould count (log cfu/g)**				
**CO (0%)**	ND	ND	1.77 ± 0.02^aA^	2.51 ± 0.10^b^
**PK (12%)**	ND	ND	1.89 ± 0.01^aB^	2.54 ± 0.02^b^

^*^Mean ± SE with different superscripts in a row (lower case alphabet) and column (upper case alphabet) differ significantly (p < 0.05), n = 6, CO = Control, PK = Pumpkin, ND = not detected.

#### Total plate count (log cfu/g)

Total plate count (TPC) increased significantly (P < 0.05) from day 0 to 21 in treated products as well as control. Similar findings were reported by Kumar et al. (2007) in chicken meat patties who also reported an increase in total plate count at each storage interval both in control and treatment samples. This is also in agreement with the findings of Chidanandaiah et al. (2009), Kumar and Tanwar (2011), Bhat et al. (2010b), (2013a) and (2013b) who also reported the similar results in meat patties, chicken nuggets, chevon *Harissa*, chicken *seekh kababs* and chicken meat balls, respectively. Between the treatment and control, the TPC were comparable (P > 0.05) on day 0, whereas TPC were significantly (P < 0.05) higher in the pumpkin treated products as the storage progressed. This could be due to easy availability of the nutrients and more favourable conditions for microbial growth. Increased extension levels and comparatively low fat percent were associated with higher counts and were in agreement with the results of Draughon (1980) who apprehended the possibility of higher microbial growth in extended foods. Harisson et al. (1983) also suggested that one limitation of extended products might be the faster spoilage rate of some extended products. Similar results were reported by Bhat et al. (2013a) and (2011) in chicken seekh kebabs extended with different non-meat proteins and Kumar et al. (2007) in chicken meat patties.

#### Psychrophillic count (log cfu/g)

Psychrophillic counts were not detected on day 0 of storage in the treated product as well as control. This could be due to the destruction of psychrophiles during cooking. But, it was observed from day 7 of the storage in treated products as well as control. The counts followed a significant (P < 0.05) increasing trend in treated as well as control products as the storage progressed. Between the treatment and control, the counts were significantly (P < 0.05) higher in treated products as compared to control. It might be due to increased possibility of ingress of microbes through pumpkin. The psychrophilic count always remained well below the maximum permissible limits in cooked meat products (Jay, 1996). Cremer and Chipley (1977) described permissible level of psychrophilic count as 4.6 log cfu/g in cooked meat and meat products. A detectable count on day 7 while nil on preceding observation might be attributed to the fact that bacteria generally need some lag phase before active multiplication is initiated. A gradual increase in psychrophilic counts during storage of chicken products had been reported by Sen and Sharma (1996), Nag et al. (1998) and Kalaikannan et al. (2007). Similar results were also reported by Bhat et al. (2013a) and (2011) in chicken seekh kebabs extended with different non-meat proteins and Kumar et al. (2007) in chicken meat patties.

#### Coliform count (log cfu/g)

The coliforms were not detected throughout the period of storage in both control and treated sausages. It could be due to the destruction of these bacteria during cooking at 80°C, much above their death point of 57°C (Jay, 1996). Further, hygienic practices followed during the preparation and packaging of products could also be one of the reasons for the absence of coliforms. Similar results were reported by Bhat et al. (2013a) in chicken *seekh kababs*, Bhat et al. (2013b) in chicken meat balls, Bhat et al. (2010b) in chevon *Harrisa*, Singh et al. (2011) in chicken snacks, Kandeepan et al. (2010) in buffalo meat *keema* and Kumar and Sharma (2004) in chicken patties who also reported zero count of coliforms for the products heated to such a high temperature.

#### Yeast and mould count (log cfu/g)

No yeast and moulds were detected up to 7^th^ day of storage both in the control as well as treated products. They were detected on day 14^th^. The counts were significantly (P < 0.05) higher in the treated product as compared to control on 14^th^ day, however, a non-significant (P > 0.05) effect was reported on day 21 of storage. The counts increased with advancement of storage days. This could possibly be due to post processing contamination and handling. Das et al. (2013) and Singh et al. (2011) also reported similar results in chicken nuggets and chicken snacks, respectively.

#### Sensory analysis

The mean values of various sensory parameters namely appearance and colour, flavour, juiciness, texture and overall acceptability of aerobically packaged chicken sausages incorporated with 0 and 12 percent levels of pumpkin during refrigerated storage (4 ± 1°C) are presented in Table 6.

**Table 6:** **Effect of refrigerated storage on sensory attributes of aerobically packaged chicken sausages (Mean ± SE)** ^*^

Treatments	Storage period (Days)			
	0	7	14	21
**Appearance and colour**				
**CO (0%)**	7.09 ± 0.09^c^	7.23 ± 0.13^c^	5.90 ± 0.11^b^	4.71 ± 0.10^a^
**PK (12%)**	7.23 ± 0.13^c^	7.19 ± 0.08^c^	6.04 ± 0.10^b^	5.04 ± 0.16^a^
**Flavour**				
**CO (0%)**	7.28 ± 0.10^d^	6.47 ± 0.22^cB^	5.38 ± 0.25^b^	4.66 ± 0.12^a^
**PK (12%)**	7.33 ± 0.14^c^	5.76 ± 0.23^bA^	5.57 ± 0.23^b^	4.56 ± 0.14^a^
**Juiciness**				
**CO (0%)**	6.85 ± 0.14^d^	6.38 ± 0.12^c^	5.38 ± 0.20^b^	4.52 ± 0.13^a^
**PK (12%)**	7.04 ± 0.16^d^	6.23 ± 0.13^c^	5.32 ± 0.21^b^	4.52 ± 0.14^a^
**Texture**				
**CO (0%)**	7.47 ± 0.11^dB^	6.52 ± 0.11^c^	5.14 ± 0.12^b^	4.28 ± 0.10^a^
**PK (12%)**	7.14 ± 0.12^dA^	6.28 ± 0.10^c^	5.09 ± 0.13^b^	4.33 ± 0.12^a^
**Overall acceptability**				
**CO (0%)**	7.66 ± 0.12^d^	6.61 ± 0.16^c^	5.42 ± 0.13^b^	4.33 ± 0.12^a^
**PK (12%)**	7.52 ± 0.20^d^	6.57 ± 0.16^c^	5.57 ± 0.18^b^	4.28 ± 0.15^a^

^*^Mean ± SE with different superscripts in a row (lower case) and column (upper case) differ significantly (p < 0.05), n = 21, CO = Control, PK = Pumpkin.

The sensory attributes were significantly affected during 21 days of refrigerated storage and all the sensory parameters viz. colour and appearance, flavour, juiciness, texture and overall acceptability followed a descending trend (P < 0.05) with increase in storage days. As the storage days progressed, all the sensory attributes followed a significant (P < 0.05) decreasing trend, however, in between treatment and control, sensory attributes were comparable (p > 0.05) throughout the storage period. The decrease in colour and appearance scores might be due to pigment and lipid oxidation resulting in non-enzymatic browning. A decrease in appearance and colour scores of meat products with increase in storage period was also reported by Bhat et al. (2013a) in chicken *seekh kababs*, Bhat et al. (2013b) in chicken meat balls, Bhat et al. (2011) in mutton *Harrisa*, Bhat et al. (2010b) in chevon *Harrisa*, Singh et al. (2011) in chicken snacks, Kandeepan et al. (2010) in buffalo meat *keema*, Chidanandaiah et al. (2009) in buffalo patties and Kilinc (2009) in anchovy patties. A gradual decline of flavour might be due to the expected loss of volatile flavour components from spices and condiments on storage of meat products. The progressive decrease in flavour could be correlated to increase in thiobarbituric acid reacting substances value of meat products (Tarladgis et al., 1960) stored under aerobic conditions. Decline in flavour scores of meat products during storage were reported by Bhat et al. (2013a) and (2011) in chicken *seekh kababs*, Bhat et al. (2013b) in chicken meat balls, Bhat et al. (2011) in mutton *Harrisa*, Bhat et al. (2010b) in chevon *Harrisa* and Thomas et al. (2006) in buffalo meat nuggets. Juiciness scores followed a significant (P < 0.05) decreasing trend in chicken sausages prepared with fibre source as well as control throughout the storage period. It could be due to some loss of moisture from the products during storage. The results were in accordance with findings of Bhat et al. (2013a), (2013b), Chidanandaiah et al. (2009) and Thomas et al. (2006). Texture scores followed a decreasing trend throughout the period of storage. However, scores were comparable in treated and control products throughout the storage period. The lower textural scores might be due to loss of water during storage and subsequent reduction of pH and denaturation of proteins at low pH and degradation of muscle fibre proteins by bacterial action (Jay, 1996), which resulted in decreased water binding capacity. Similar results were presented by Bhat et al. (2013a) and (2011) in chicken *seekh kababs*, Bhat et al. (2013b) in chicken meat balls, Kilinc (2009) in anchovy patties and Thomas et al. (2006) in buffalo meat nuggets during refrigerated storage, respectively. The overall acceptability scores of both control and the treated sausages decreased significantly (P < 0.05) as the storage progressed. This decrease might be reflective of the decline in scores of flavour, juiciness and texture attributes. Similar findings have also been reported by Kumar and Sharma (2004), Bhat and Pathak (2009), Bhat et al. (2013a) and (2013b) for various meat products. These observations indicated that chicken sausages prepared with 12 percent pumpkin retained good to very good sensory attributes up to day 14 under refrigerated storage at 4 ± 1°C in low density polyethylene (LDPE) pouches.

## Conclusions

The present study showed successful utilization of pumpkin in the preparation of chicken sausages and the products had almost similar sensory attributes and acceptability as in control sausages. Thus, chicken sausages of good to very good acceptability and nutritive value could be prepared by incorporating pumpkin pulp substituting lean chicken meat in formulation by oven-roasting method of cooking. The chicken sausages extended with pumpkin pulp could be conveniently packed in LDPE for a period of 14 days in refrigerated (4 ± 1°C) condition without any marked loss of physicochemical, microbial and sensory quality. Thus, comparatively low-cost fibre enriched chicken sausages were developed utilizing chicken meat and further replacing the same with suitable vegetable fibre. This study would also help in utilization of pumpkin in other meat products. Further research should be focused on the use of pumpkin in other meat products particularly in dehydrated powder form.
